# The potential crosstalk genes and molecular mechanisms between glioblastoma and periodontitis

**DOI:** 10.1038/s41598-024-56577-2

**Published:** 2024-03-12

**Authors:** Jian-huang Huang, Yao Chen, Yuan-bao Kang, Zheng-jian Yao, Jian-hua Song

**Affiliations:** Department of Neurosurgery, Affiliated Hospital of Putian University, Putian, Fujian China

**Keywords:** Glioblastoma, Periodontitis, Crosstalk genes, Immune infiltration, Bioinformatics analysis, Cancer microenvironment, CNS cancer, Head and neck cancer

## Abstract

Despite clinical and epidemiological evidence suggestive of a link between glioblastoma (GBM) and periodontitis (PD), the shared mechanisms of gene regulation remain elusive. In this study, we identify differentially expressed genes (DEGs) that overlap between the GEO datasets GSE4290 [GBM] and GSE10334 [PD]. Functional enrichment analysis was conducted, and key modules were identified using protein–protein interaction (PPI) network and weighted gene co-expression network analysis (WGCNA). The expression levels of CXCR4, LY96, and C3 were found to be significantly elevated in both the test dataset and external validation dataset, making them key crosstalk genes. Additionally, immune cell landscape analysis revealed elevated expression levels of multiple immune cells in GBM and PD compared to controls, with the key crosstalk genes negatively associated with Macrophages M2. FLI1 was identified as a potential key transcription factor (TF) regulating the three key crosstalk genes, with increased expression in the full dataset. These findings contribute to our understanding of the immune and inflammatory aspects of the comorbidity mechanism between GBM and PD.

## Introduction

Glioblastoma (GBM) is a highly aggressive primary brain tumor and one of the deadliest and most recurrent solid tumors^1^, accounting for 57% of all gliomas and 48% of primary central nervous system malignancies^2^. Despite comprehensive treatment regimens including surgery, chemotherapy, and radiotherapy, the median survival of GBM patients after diagnosis is only about 15 months, with a 5-year survival rate of less than 10%^3^. Therefore, investigating the pathogenesis of GBM, particularly the study of modifiable risk factors for GBM progression, is crucial to improve the prognosis of GBM patients.

Periodontitis (PD), primarily caused by *Porphyromonas* *gingivalis*, is a chronic inflammatory disease marked by the gradual destruction of periodontal tissue^4^. During the active phase of PD, *Porphyromonas* *gingivalis* partially breaches the periodontal tissue, enters the bloodstream, and causes bacteremia. This leads to the release of numerous inflammatory mediators, ultimately provoking a prolonged low-grade inflammatory response in distant organs^5^. Chronic inflammation is widely believed to be associated with tumor formation and progression. Studies indicate that periodontitis elevates the risk of oral^6^, liver^7^, and colorectal cancers^8^ and is correlated with an increased overall cancer mortality rate^9^. In recent years, research has increasingly focused on the interaction between glioblastoma (GBM) and periodontitis (PD). Investigative studies have found that glioma patients exhibit significantly poorer periodontal health and a higher incidence of periodontitis compared to the general population^10^. Experimental studies further show that *Porphyromonas* *gingivalis* or its lipopolysaccharide (LPS) can cross the blood–brain barrier, enter brain tissue, and stimulate the proliferation and migration of glioma cells at varying concentrations^11^. *Porphyromonas* *gingivalis* is not only linked to glioma grading but also shows a significant correlation with IDH1 (isocitrate dehydrogenase 1) mutations in gliomas^12^. These mutations are crucial prognostic factors in glioma patients. These findings indicate that periodontal pathogens may have a significant role in glioma development. Nevertheless, the precise mechanisms through which periodontitis contributes to the initiation and progression of glioblastoma remain incompletely understood.

In this study, we utilized public datasets from the GEO database and TCGA-GBM to conduct bioinformatics analyses including differential expression gene (DEG) identification, functional enrichment analysis, weighted gene co-expression network analysis (WGCNA), and the Cibersort algorithm. The aim of this study is to explore the common pathogenesis of glioblastoma (GBM) and periodontitis (PD), to provide new insights and approaches for controlling the development and progression of gliomas, as well as for the treatment of glioblastoma.

## Materials and methods

### Data sources

**Figure 1:**
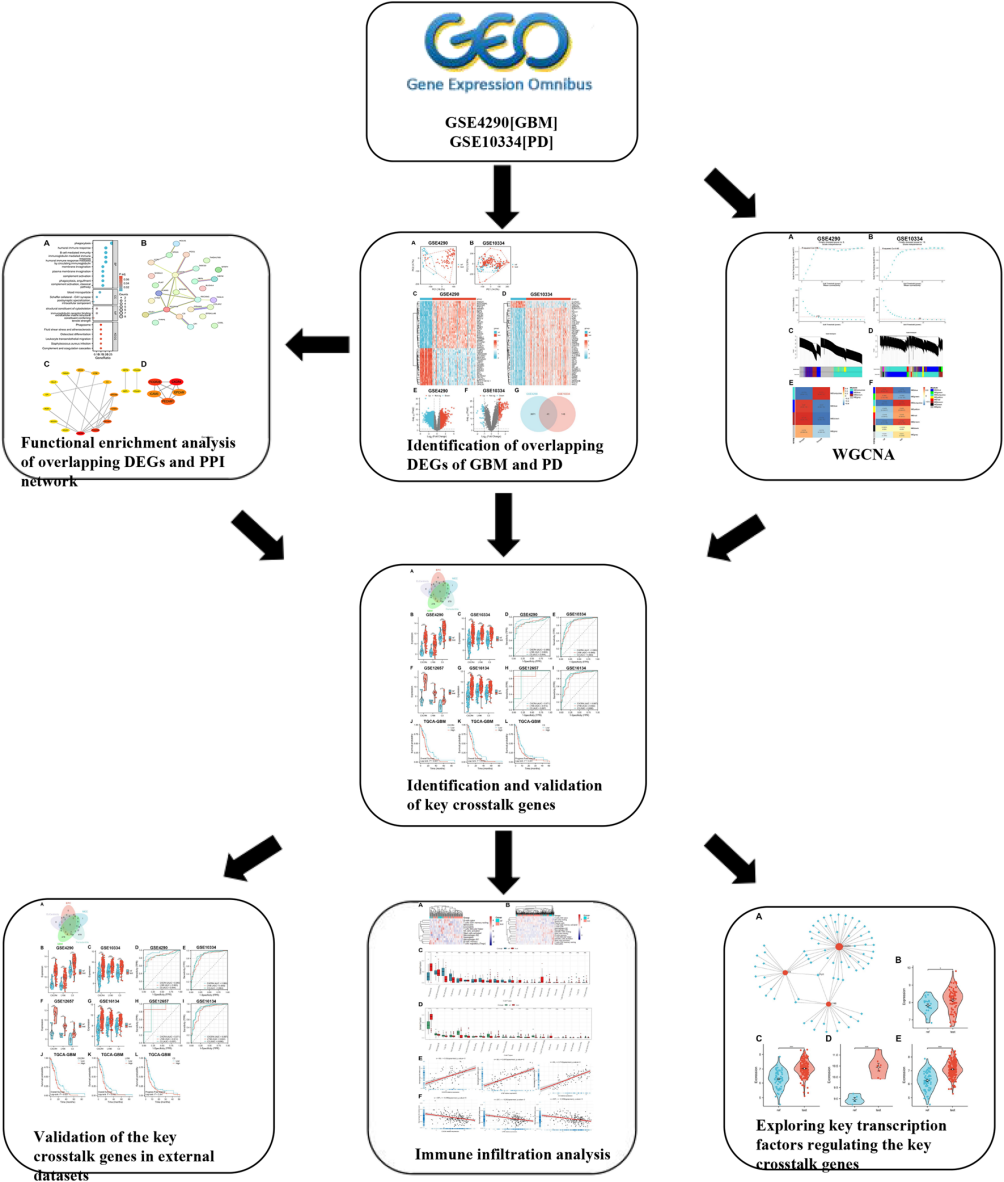
The flow diagram for the whole study.

The study's flowchart is depicted in Fig. 1. The expression data for GBM and PD were obtained from the Gene Expression Omnibus (GEO) database (https://www.ncbi.nlm.nih.gov/geo). The search strategy for the GEO dataset included the following: (1) topic search for “glioblastoma” and “periodontitis,” respectively; (2) study type selection as “Expression profiling by array”; (3) samples were derived from Homo sapiens; and (4) the dataset contained normal control group samples. Based on the study design, the GEO datasets GSE4290 [GBM] and GSE10334 [PD] were used as the test set, with the former retaining only 23 normal samples and 81 GBM samples, and the latter containing 183 PD-affected gingival tissue samples and 64 unaffected gingival tissue samples.

### Differentially expression analysis

The preliminary analyses of the dataset in this study were conducted using R software (version 4.2.0). The required disease and control samples were extracted according to the research needs. If the expression data had not been log2 transformed, it was manually log2 transformed. To identify the differentially expressed genes (DEGs) using the ‘Limma’ package^13^, the expression levels were normalized using the ‘normalizeBetweenArrays’ function to eliminate batch differences. Genes with a |log2 fold change| > 1 and an adjusted P-value < 0.05 were identified as DEGs. Overlapping DEGs, which were up-regulated or down-regulated in both sets of DEGs, were defined. Venn plots of overlapping DEGs were created using the “ggvenn” package.

### Enrichment analysis of overlapping DEGs

To analyze the overlapping DEGs, we employed the “clusterProfiler” package to conduct Gene Ontology (GO) and Kyoto Encyclopedia of Genes and Genomes (KEGG) enrichment analyses^14,15^. Only terms with an FDR < 0.05 were considered to be significantly enriched.

### Protein–protein interaction (PPI) network construction

To construct a PPI network with overlapping DEGs, we utilized the STRING database V11.5 (available at http://string-db.org). We set the “minimum required interaction score” parameter to 0.4 to exclude unconnected nodes. The resulting PPI network was then imported into Cytoscape software V3.9.1^16^ for visualization. To filter clusters with high connectivity, we employed the MCODE plug-in and used default parameters to divide the PPI network into multiple clusters. The CytoHubba plugin utilizes eleven node ranking methods to assess the significance of nodes within a biological network. These methods comprise Degree, Edge Percolated Component, Maximum Neighborhood Component, Density of Maximum Neighborhood Component, Maximal Clique Centrality, Bottleneck, EcCentricity, Closeness, Radiality, Betweenness, and Stress^17^. To provide a comprehensive evaluation of these genes, we employed both local methods (MCC) and global methods (EPC and EcCentricity). Local methods solely focus on the relationship between a node and its immediate neighbors, whereas global methods assess the node’s relationship with the entire network. Finally, we analyzed the genes within the clusters for functional enrichment.

### Weighted gene co-expression network analysis (WGCNA)

The top 5000 genes with the highest absolute median difference in expression in the test dataset were screened for WGCNA analysis using the “WGCNA” package^18^. To begin, a soft threshold was obtained using the pickSoftThreshold function. Next, a weighted adjacency matrix was constructed. Finally, the correlation between each module and the disease was calculated. The module with the highest correlation with the grouped traits was identified as the key module.

### Identification and validation of key crosstalk genes

Firstly, we utilized Cytoscape software's CytoHubba plugin^17^ to identify the hub genes in the PPI network. The genes that were ranked in the top 10 using various algorithms were determined as the PPI key genes. Subsequently, the PPI key genes were intersected with the key module genes of WGCNA, and these intersected genes were designated as the key crosstalk genes. We then compared the mRNA expression levels of the key crosstalk genes between the case and control groups using the Mann–Whitney *U* test. The results were considered statistically significant if the P value was less than 0.05, which was visualized using the “ggplot2” package. Finally, we evaluated the diagnostic efficacy of the key crosstalk genes in the test dataset by constructing receiver operating characteristic (ROC) curves using the “pROC” package.

### Validation of key crosstalk genes in an independent external dataset

To validate the mRNA expression levels of key crosstalk genes, we utilized independent external datasets GSE14805 [GBM] and GSE16134 [PD]. In this study, GSE14805 included 4 control samples and 34 GBM samples. GSE16134 contained 241 PD-affected gingival tissue samples and 69 unaffected gingival tissue samples.

### Immune infiltration analysis

First, immune cell expression levels in the test dataset were analyzed in the case and control groups using the “cibersort” algorithm^19^. Then, we calculated the correlation between key crosstalk gene expression and immune cell expression. We used the Spearman method to calculate the correlation coefficient and P-value between genes and immune cells. P < 0.05 was considered statistically significant and was visualized by “ggplot2”.

### Identification of transcription factors (TFs) of key crosstalk genes

The TFs of key crosstalk genes were predicted by NetworkAnalyst 3.0 (available at https://www.networkanalyst.ca). We compared the average expression levels of these TFs in samples between different groups using the Mann–Whitney *U* test in both the test set and validation set. The results showed that TFs that were usually upregulated in the case group were identified as potential key TFs in GBM and PD.

## Results

### Identify overlapping DEGs of GBM and PDs

**Figure 2:**
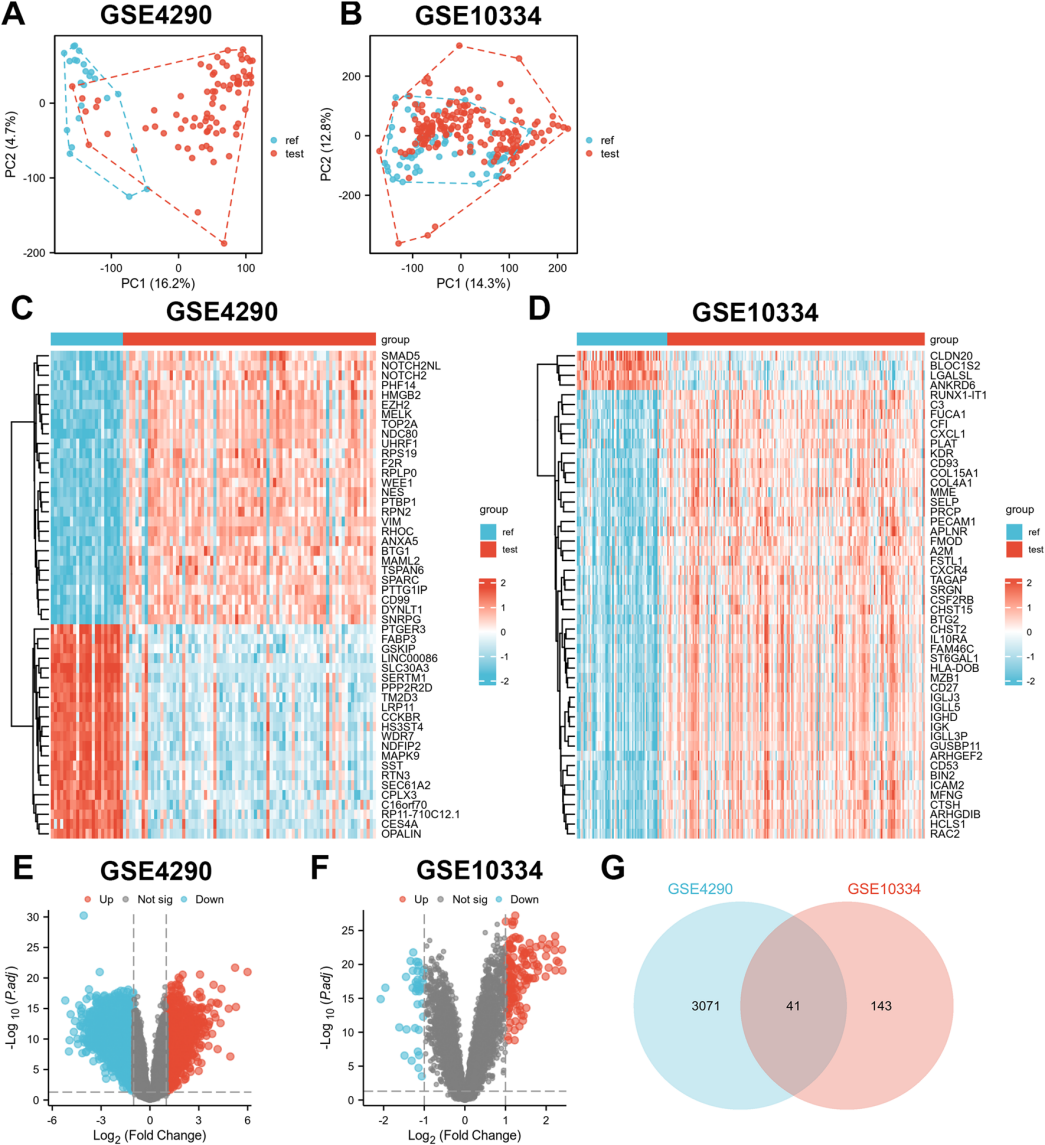
DEGs of test datasets GSE4290 and GSE10334. (**A**), (**C**), and (**E**) are PCA plots, heat maps, and volcano plots of GSE4290 differential gene analysis, respectively. (**B**), (**D**), and (**F**) are PCA plots, heat maps, and volcano plots of GSE10334 DEG analysis, respectively. (**G**) DEGs of GSE4290 and GSE10334 were crossed to obtain 41 crossover DEGs.

This study obtained satisfactory expression data through data preprocessing and normalization (Supplementary Fig. 1). PCA was used to downscale the mRNA expression data of the two test datasets, revealing significant differences between the case and control groups with good sample homogeneity (Fig. 2A–D). The “Limma” R package identified 3112 DEGs in dataset GSE4290, of which 1193 were up-regulated and 1919 were down-regulated (Fig. 2E). In dataset GSE10334, the “Limma” R package identified 184 DEGs, of which 146 were up-regulated and 38 were down-regulated (Fig. 2F). Subsequently, intersecting DEGs were identified (Fig. 2G), and the expression trends of these genes were examined, resulting in the identification of 30 overlapping DEGs.

### Functional enrichment analysis of overlapping DEGs

**Figure 3:**
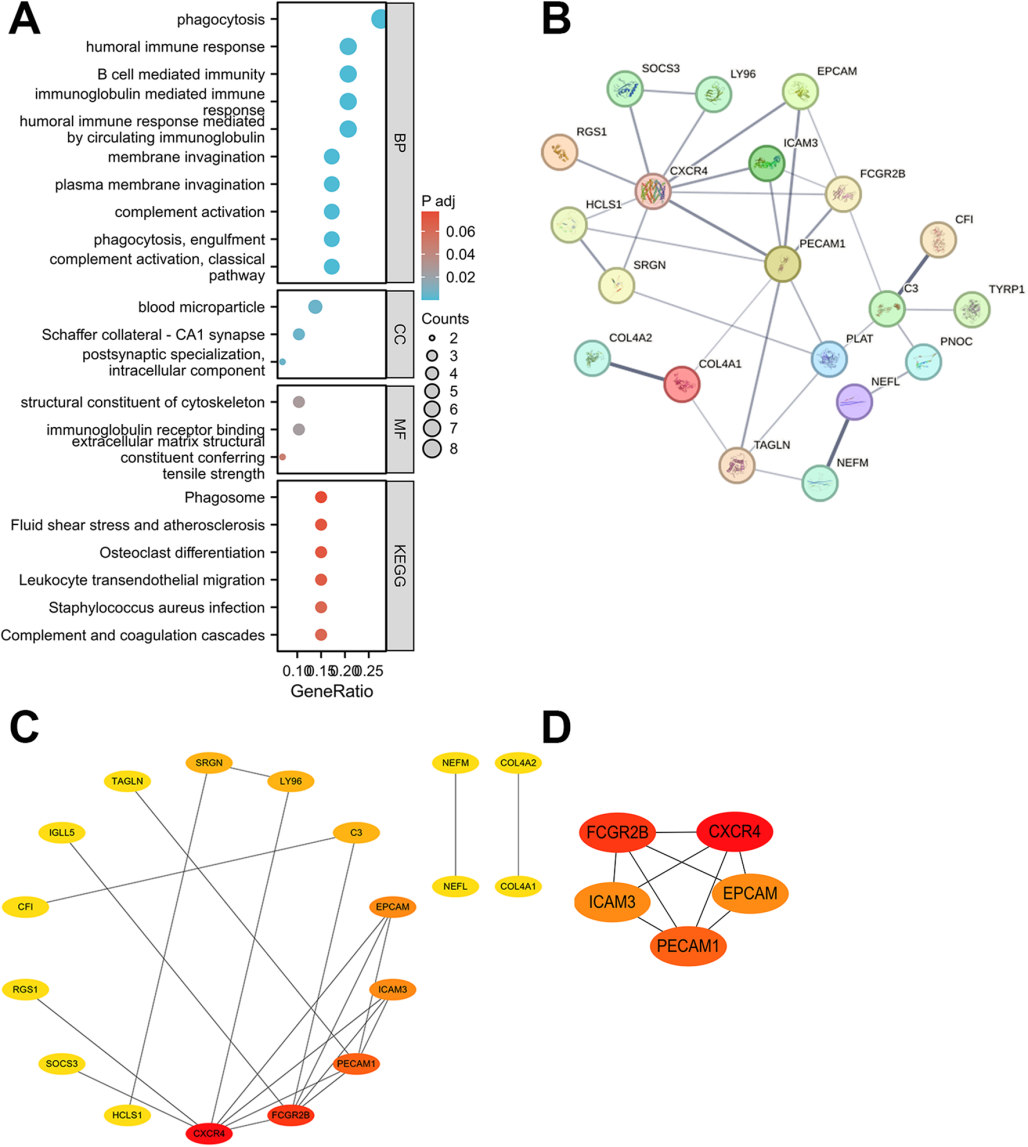
Functional enrichment analysis of overlapping DEGs and PPI network construction for the test dataset. (**A**) Functional enrichment analysis of overlapping DEGs. (**B**) PPI network constructed by overlapping DEGs. The thickness of the line represents the interaction intensity, and the thicker it is, the greater the interaction intensity. (**C**) Network construction of overlapping DEGs by Degree algorithm. The redder the color, the larger the Degree value. (**D**) PPI network obtained by Mcode algorithm for cluster 1.

We performed a functional enrichment analysis of the overlapping DEGs (Fig. 3A). Gene Ontology Biological Process (GO_BP) analysis revealed that the most significantly enriched terms were phagocytosis, humoral immune response-mediated circulating immunoglobulins, complement activation (classical pathway), immunoglobulin-mediated immune response, B cell-mediated immunity, and phagocytosis. KEGG analysis revealed that overlapping DEGs may be associated with complement and coagulation cascades, *Staphylococcus* *aureus* infection, leukocyte transendothelial migration, osteoclast differentiation, fluid shear stress, atherosclerosis, and phagosome. Therefore, it is clear that the functions of overlapping DEGs are strongly associated with immune and inflammatory processes.

### Building PPI networks with overlapping DEGs

A PPI network with 28 nodes and 32 edges (PPI enrichment P-value < 1.5e−11) was constructed by entering the overlapping DEGs into the STRING database (Fig. 3B). The PPI network was visualized using Cytoscape software (Fig. 3C). A cluster with high connectivity was identified by the Mcode plug-in (Fig. 3D). This cluster contained 5 nodes, 9 edges and a score of 4.500. The genes included are CXCR4, EPCAM, FCGR2B, PECAM1, and ICAM3.

### WGCNA construction and key module screening

**Figure 4:**
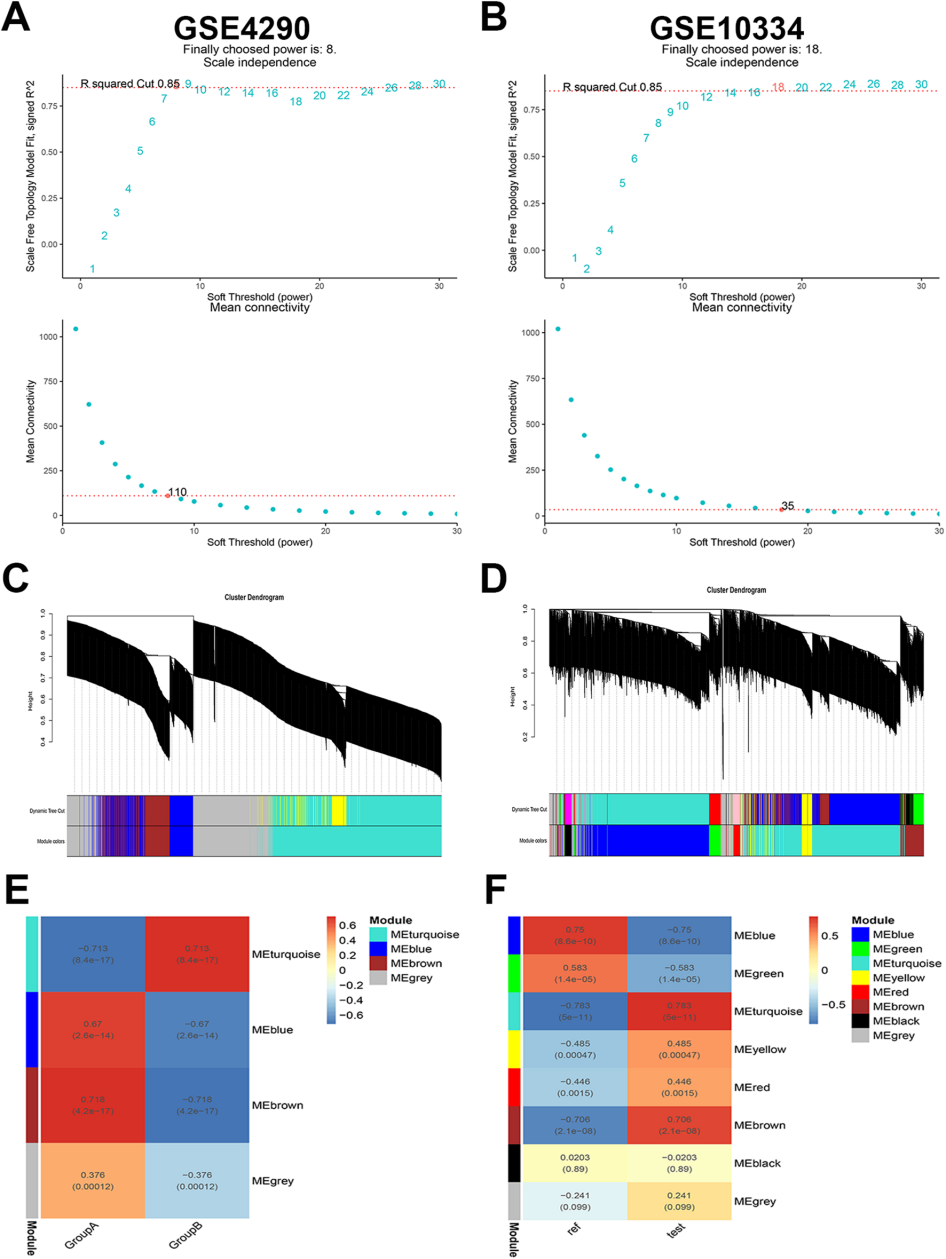
Weighted gene co-expression network analysis (WGCNA) of the test dataset. (**A**,**B**) Soft threshold selection for GSE4290 and GSE10334. (**C**,**D**) Clustering dendrogram of the top 5000 genes with the highest absolute median difference in expression between GSE4290 and GSE10334 based on differential measures. (**E**,**F**) Module-trait relationship between GSE4290 and modules of GSE10334 in relation to traits. Different colors represent different modules and contain the corresponding correlations and P-values.

WGCNA analysis was performed using the top 5000 genes with the highest absolute median difference in expression in the test dataset. β = 8 and β = 18 (scale-free R2 = 0.85) were chosen as soft thresholds for GBM and PD, respectively, to ensure scale-free networks (Fig. 4A,B). WGCNA analysis identified 4 modules in GSE4290 and 8 modules in GSE10334 (Fig. 4C,D). Finally, a heat map of module-trait relationships was generated based on Pearson correlation coefficients. The results showed that the brown module had the highest correlation with GBM (0.718, P = 4.2E−17) and contained 272 genes; the turquoise module had the highest correlation with PD (0.783, P = 5E−11) and included 705 genes (Fig. 4E,F).

### Identification and validation of key crosstalk genes

**Figure 5:**
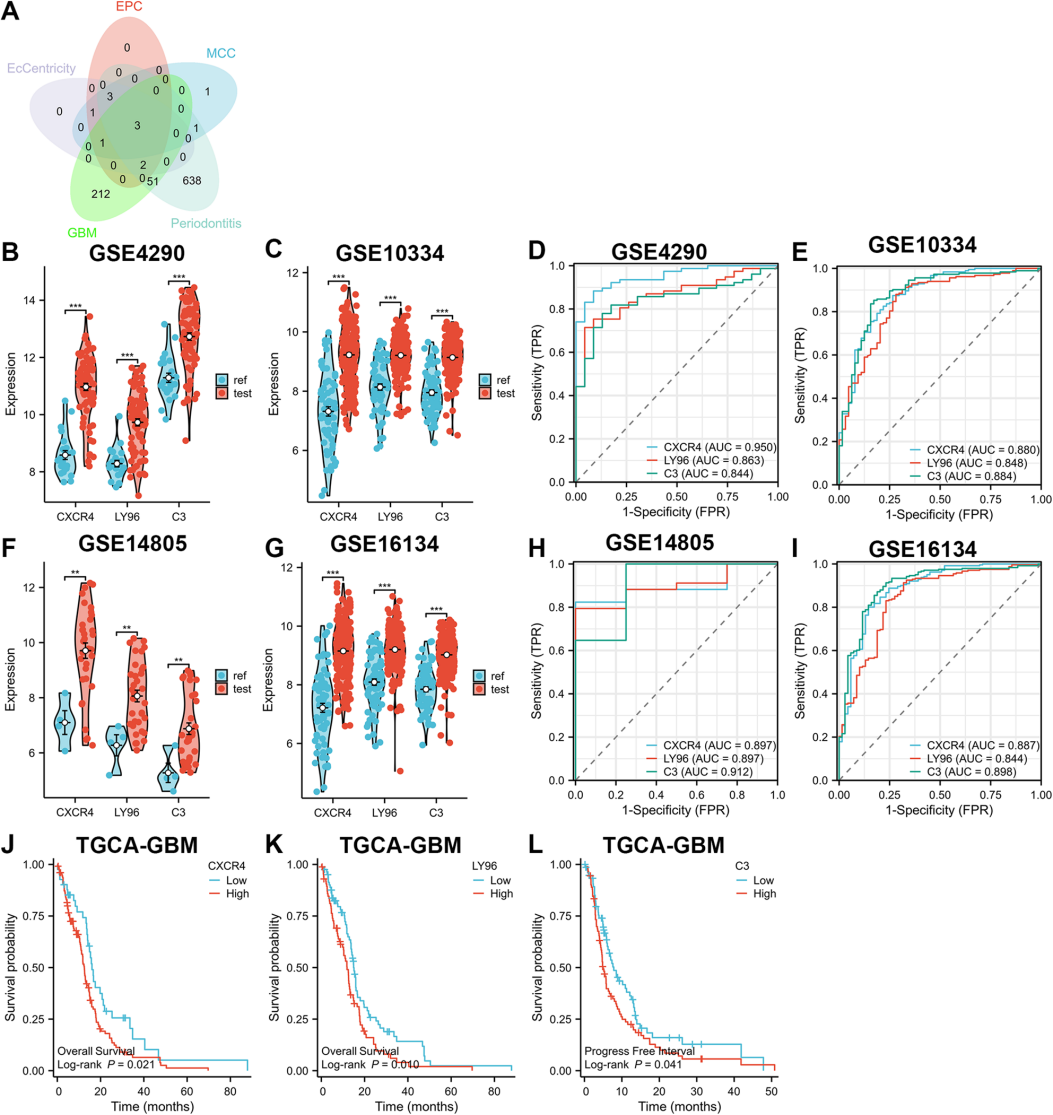
Identification and validation of key crosstalk genes. (**A**) CXCR4, LY96 and C3 mRNA are present in all three algorithms and two WGCNA key modules. (**B**,**C**) In the test set, CXCR4, LY96 and C3 mRNA expression levels are significantly higher in the case group than in the control group. (**D**,**E**) In the test set, CXCR4, LY96 and C3 have good diagnostic ability for GBM and PD. (**F**,**G**) In the validation set, CXCR4, LY96 and C3 mRNA expression levels were significantly higher in the case group than in the control group. (**H**,**I**) In the validation set, CXCR4, LY96 and C3 were diagnostically good for GBM and PD. (**J**–**L**) The TGCA-GBM dataset explores the prognosis of CXCR4, LY96 and C3 and GBM relationship. ***P < 0.0001. *P < 0.01.

We employed the CytoHubba plugin to sort the top 10 genes under the Maximal Clique Centrality (MCC), Edge Percolated Component (EPC) and Eccentricity (EC) algorithms^22^. The Venn diagram revealed that CXCR4, LY96, and C3 were present in both the top 10 genes of the three algorithms and in the two key modules (Fig. 5A). Consequently, these three genes were identified as key crosstalk genes. In GSE4290 and GSE10334, mRNA expression levels of CXCR4, LY96, and C3 were significantly higher in the case group than in the control group (Fig. 5B,C). Moreover, CXCR4, LY96, and C3 exhibited satisfactory diagnostic ability for GBM and PD based on ROC curves, with AUC values exceeding 0.8 (Fig. 5D,E).

### Validation of key crosstalk genes in an independent external dataset

To increase the confidence level, we validated the expression of key crosstalk genes in two other independent external datasets (GSE14805 [GBM] and GSE16134 [PD]). The expression differences were consistent with the test set (Fig. 5F,G), while demonstrating good diagnostic efficiency (Fig. 5H,I). Subsequently, we analyzed using the TGCA-GBM dataset and discovered that high expression of CXCR4, LY96, and C3 was associated with poor prognosis of GBM (Fig. 5J–L).

### Immuno-infiltration analysis

**Figure 6:**
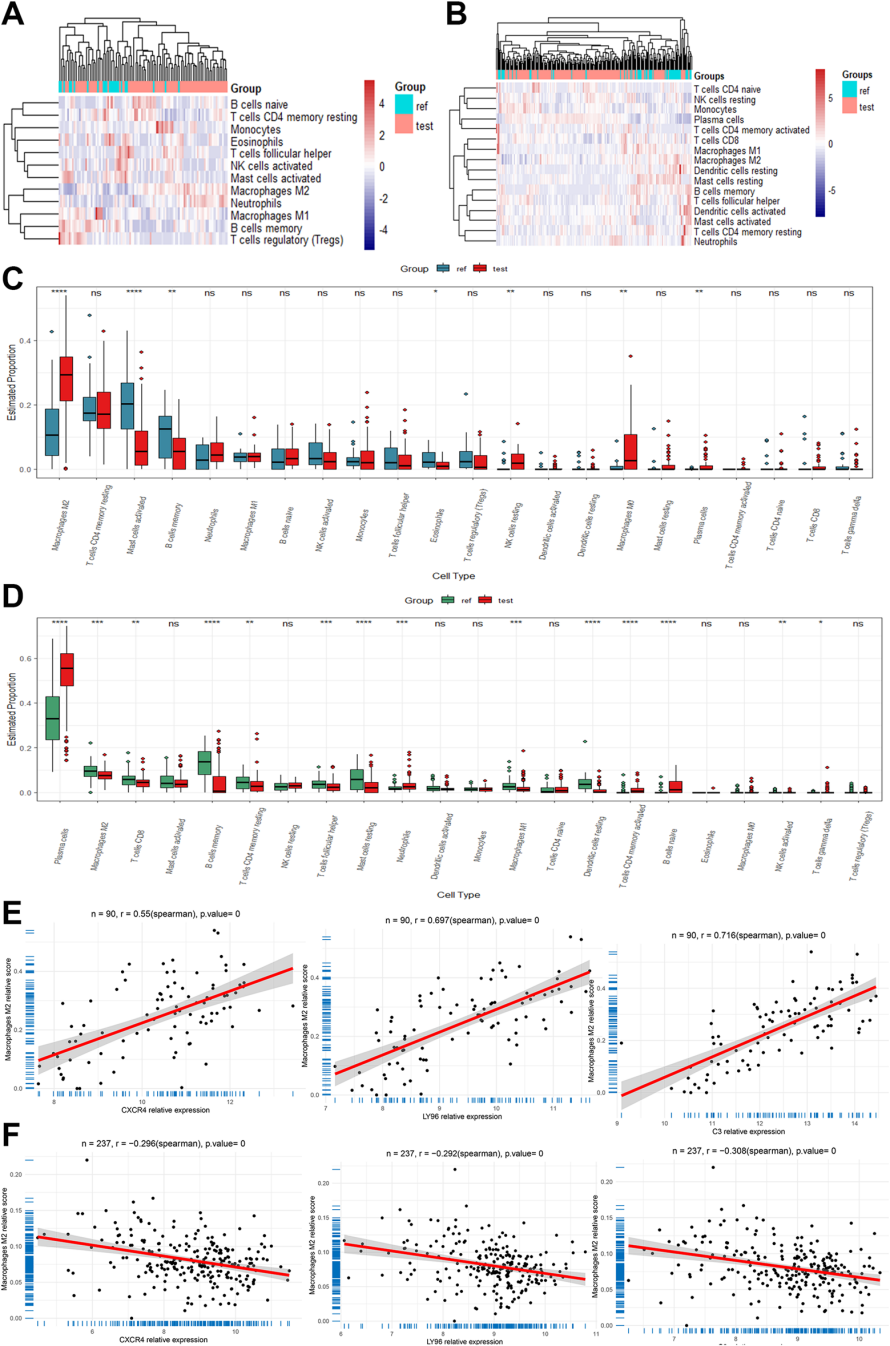
Immune infiltration analysis of the test dataset. (**A**,**B**) Immune landscape between groups in dataset GSE4290 and GSE10334. (**C**) Comparison of immune cell composition between case groups and controls in dataset GSE4290. (**D**) Comparison of immune cell composition between case groups and controls in dataset GSE10334. (**E**) CXCR4, LY96 and C3 expression levels in dataset GSE4290 are significantly positively correlated with Macrophages M2, LY96 and C3 expression levels were significantly and positively correlated with Macrophages M2 in dataset GSE4290. (**F**) CXCR4, LY96 and C3 expression levels were significantly and negatively correlated with Macrophages M2 in dataset GSE10334. ***P < 0.0001; **P < 0.001; *P < 0.01.

By conducting an enrichment analysis of overlapping DEGs, we found that immune and inflammatory processes are involved in the crosstalk between GBM and PD. Therefore, we utilized the cibersort algorithm to analyze the proportion of immune cells in the case group versus control samples in both test datasets. We found a significantly altered immune landscape in GBM and PD in the case group, with a reciprocal trend in Macrophages M2 (Fig. 6A–D). Correlation analysis demonstrated that CXCR4, LY96, and C3 expression levels were significantly and positively correlated with Macrophages M2 in GBM (Fig. 6E), and the opposite in PD (Fig. 6F). Subsequently, we further validated the above results using an external validation set (supplementary materials—Fig. 2).

### Exploring key transcription factors (TFs) that regulate key crosstalk genes

**Figure 7:**
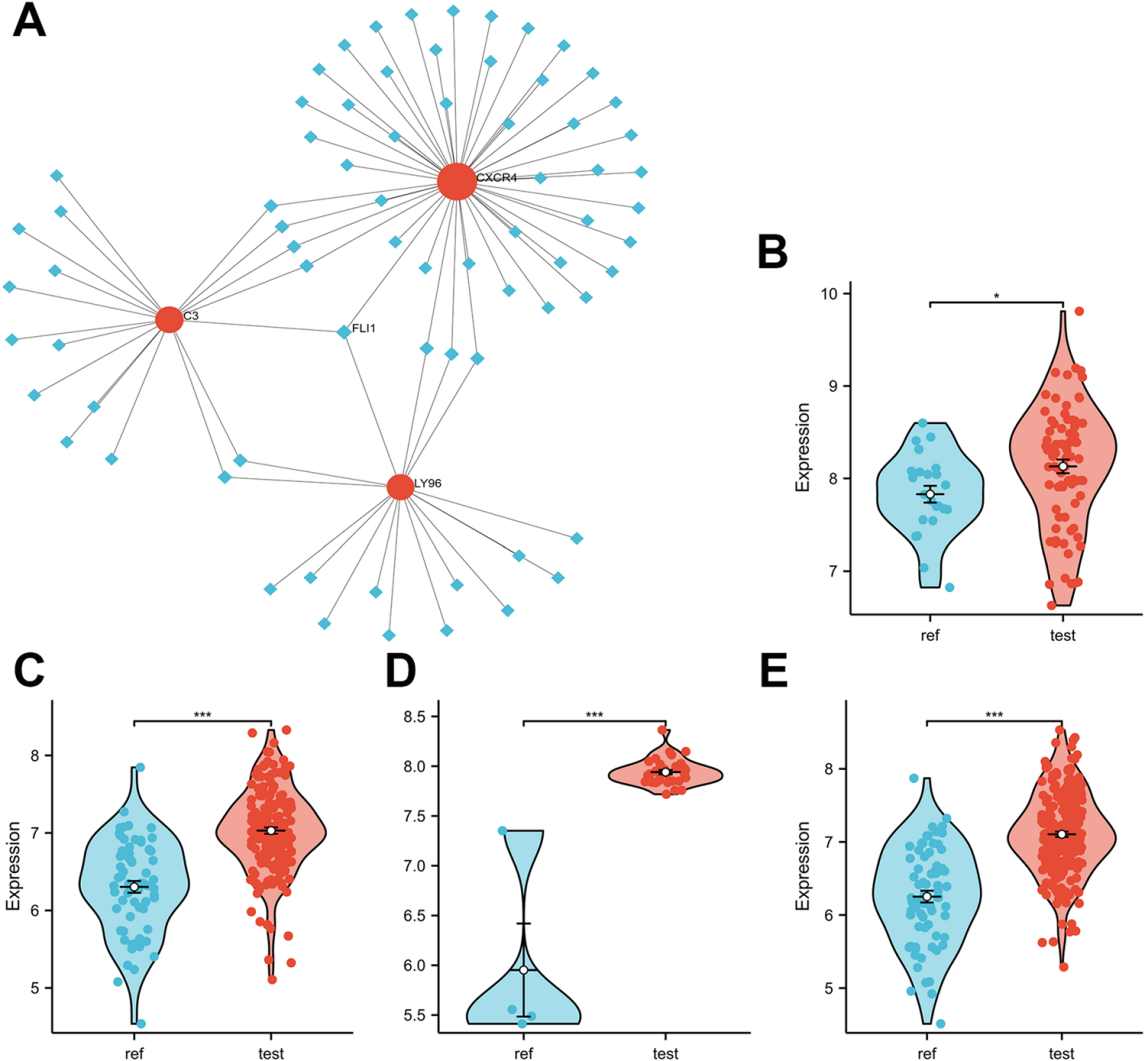
FLI1 is identified as a potential key TF shared in GBM and PD. (**A**) NetworkAnalyst 3.0 exploration suggests that FLI1 regulates both CXCR4, LY96 and C3 genes. (**B**,**C**) FLI1 was significantly elevated in the test dataset (GSE4290 and GSE10334) case set. (**D**,**E**) FLI1 was significantly elevated in the external validation dataset (GSE14805 and GSE16134) case set. *P < 0.01; **P < 0.001; ***P < 0.0001.

Furthermore, we examined potential transcription factors that may regulate CXCR4, LY96, and C3 genes using NetworkAnalyst 3.0 and compared the expression levels of case and control groups in all datasets using the Mann–Whitney *U* test. We discovered that FLI-1 interacted with CXCR4, LY96, and C3 simultaneously (Fig. 7A) and that FLI-1 expression levels were significantly elevated in all case groups (Fig. 7B–E). Therefore, FLI-1 may be a potential key transcription factor regulating two crucial crosstalk genes in the pathological process of GBM and PD.

## Discussion

Both GBM and PD are prevalent health issues that often impose a significant burden on families and society. Previous reports have suggested a strong association between GBM and PD^11^. However, the specific pathogenic mechanism and crosstalk genes remain unclear. In this study, we conducted a bioinformatics analysis of the crosstalk between GBM and PD. Our findings revealed that CXCR4, LY96, and C3 genes play a crucial role in the co-pathogenesis of both diseases. Furthermore, we explored the transcription factors of crosstalk genes and discovered that Friend leukemia integration 1 (FLI1) regulates the above three crosstalk genes simultaneously. This study provides a new direction for future research on the relationship between periodontitis and the onset and progression of GBM as well as targeted therapy. It is worth noting that this study may be the first literature to report crosstalk genes between GBM and PD.

Chemokine (C-X-C motif) receptor 4 (CXCR4), a G protein-coupled receptor, binds its typical ligand stromal cell-derived factor 1 (SDF-1). Although CXCR4 signaling is essential for individual development and organ repair^20^, high CXCR4 expression has been linked to an increased risk of cancer^21^. The present study found that CXCR4 is overexpressed in GBM and is associated with a poorer prognosis^22^, consistent with previous findings. In GBM, ligand binding to CXCR4 induces conformational changes that activate PI3K-AKT, JAK/STAT, and MEK1/2-Erk1/2 pathways, leading to the activation of STAT3, an oncogenic transcription factor involved in GBM growth^23^. Inhibition of MEK-ERK1/2 signaling enhances the adhesion of glioma cells to the ECM and reduces cell proliferation and migration^24^. Additionally, CXCR4 was found to be overexpressed in periodontitis gingival tissue, as shown in this study^25^. Activation of CXCR4 by *Porphyromonas* *gingivalis* leads to crosstalk with Toll-like receptor 2 (TLR2), which disrupts the killing function of monocytes or macrophages by inhibiting NO production and increasing cAMP-dependent protein kinase A (PKA) signaling^26^. Furthermore, PI3K-dependent adhesion pathway activation via CXCR4 in monocytes and macrophages leads to CR3 activation, which is utilized by *Porphyromonas* *gingivalis* and other pathogens as a safe entry portal to enhance their intracellular survival^27^.

Lymphocyte antigen 96 (LY96, MD2) is a crucial component required for the activation of TLR4 by LPS in the outer wall of *Porphyromonas* *gingivalis*. It serves as the first line of defense against microbial infection^28^. LY96 expression is significantly elevated in gingival tissues of patients with periodontitis^29^, resulting in the formation of TLR4-LY96-CD14 complexes that trigger the MyD88 pathway, leading to the production of tumor necrosis factor α, IL-6, IL-8, and IL-2^30^. Recent studies have shown that LY96 is closely associated with tumorigenesis and progression in various cancers, including colon cancer^31^, and GBM^32^, with the highest expression being observed in GBM and predicting a worse prognosis^33,34^. In GBM, TLR4 is usually expressed on glioma tissues and microglia/macrophages^35^. The stimulation of TLR4/MD2 complex signaling by LPS may involve loss or mutation of the tumor suppressor PTEN^36^, which can have a significant impact on cancer susceptibility and tumorigenesis, even with subtle changes in its function.

Complement C3 is a central component where classical, lectin, and alternative pathways converge, producing effector molecules such as C3a and C5a that activate C3aR and C5aR, respectively, leading to leukocyte mobilization and activation^37^. Histological observations indicate that C3-activated complement fragments are abundant in the gingival crevices of periodontitis and positively correlate with inflammatory indices. After treatment of periodontitis, complement C3 levels significantly decrease (Top 5% genes), whereas they are absent or present at lower concentrations in healthy individuals^37^. Mechanistically, C3 activation may promote periodontal inflammation mainly by increasing vascular permeability and chemotactic recruitment of inflammatory cells through activation of C5aR increasing vascular permeability and flow of inflammatory exudates and chemotactic recruitment of inflammatory cells^38^, but it does not control infection^39^. Therefore, C3 is currently identified as one of the 21 most promising candidate genes for periodontitis treatment^40^. High levels of complement-activating proteins may be beneficial for tumors^41^. A study showed that C3 deposition was observed in GBM tissues, indicating local complement activation in GBM, and confirmed the protective effect of complement C3 on GBM development and progression^42^. It is important to note that glioma stem cells (GCS) may activate C3 with the help of alternative pathways and activate STAT-3, ERK2/1, and PI3K/Akt/mTOR pathways to maintain their pluripotent state^42^. Additionally, hypoxic conditions contribute to C3 activation and enhance C3a-C3aR effects^43^, forming an additional effector mechanism for GSC survival, self-renewal, and tumor growth.

In our study, we identified characteristic genes shared by GBM and periodontitis that were closely associated with the immune process. Firstly, based on comprehensive bioinformatics analysis, we screened overlapping DEGs of GBM and PD mRNA expression profiles and found that these genes are mainly involved in complement and coagulation cascades, *Staphylococcus* *aureus* infection, leukocyte transendothelial migration, osteoclast differentiation, fluid shear stress, atherosclerosis, phagosome, and other immune-related biological pathways. Subsequently, we identified three crosstalk genes (CXCR4, LY96, and C3) in GBM and PD using a combined screen of PPI network, hub genes, and WGCNA analysis. It is worth emphasizing that all three genes are immune-related. The ROC analysis suggested that all three crosstalk genes showed high sensitivity and specificity, which was conclusively validated in the external validation dataset. Finally, we analyzed the immune characteristics of GBM and PD using immune infiltration. The study showed decreased macrophage M2 polarization in PD, which negatively correlated with key crosstalk gene expression and was consistent with a pro-inflammatory manifestation of PD^44^. Increased macrophage M2 polarization in GBM positively correlated with key crosstalk gene expression and was consistent with an immunosuppressive tumor microenvironment in GBM^45^. We speculate that the disparities in gene effects may primarily be attributed to the distinct cells in which interacting genes function and the secretion of various inflammatory factors. For instance, in GBM cells, the upregulation of CXCR4 recruits glioma-associated microglia/macrophages (GAMs) and results in M2 polarization of macrophages^46^. Conversely, in periodontal inflammation, the upregulation of CXCR4 inhibits TLR4-induced NF-κB activation through the LPS-CXCR4 axis, thereby suppressing M2 polarization of macrophages^47^. Furthermore, it is also associated with the lack of other immune cell types, such as insufficiently activated CD8^+^ T cells and functionally impaired microglia in GBM^48,49^.

These findings suggest that these shared DEGs may bridge the common pathogenesis of GBM and PD by affecting immune cells. We further explore the mechanism of crosstalk between PD and GBM. Firstly, *Porphyromonas* *gingivalis*, the primary causative agent of PD, activates complement C3 or CXCR4, impairing the killing capacity of neutrophils and macrophages but failing to control infection^37,39,50^. This is consistent with the results of functional enrichment analysis such as phagocytosis, complement activation (classical pathway), etc. As a result, *P*. *gingivalis* proliferates uncontrollably in an inflammatory environment. Secondly, *P*. *gingivalis* disseminates distantly with the bloodstream, this conforms to the results of functional enrichment analysis of Leukocyte transendothelial migration, Cell adhesion molecules, and others. Then, LPS binding to LY96 causes disruption of the blood–brain barrier, triggering chronic and insidious inflammation formation in the brain, promoting glial cell carcinogenesis and exacerbating tumor growth^51^. Specific mechanisms include loss/mutation of the tumor suppressor PTEN^36^, maintenance and survival of GCS stemness^42^, and treatment resistance^52^. Although glioma-associated microglia and macrophages (GAMs) and MDSC are recruited into the glioma microenvironment and release various growth factors and cytokines, abnormal expression of CXCR4 and C3 leads to immunosuppression^53^, resulting in restricted clearance of cancer cells.

To investigate the candidate regulatory mechanisms of GBM and PD crosstalk genes, we constructed a target gene-TF network. Our study revealed that Friend leukemia integration 1 transcription factor (FLI1) interacts closely with three crosstalk genes simultaneously. Currently, little is known about the role of FLI1 in GBM and PD. Only literature shows that FLI1 is overexpressed in GBM tissues and enhances GBM radiotherapy resistance^54^. This finding is consistent with the results of our present study. However, further validation is needed in PD.

The current study identified key crosstalk genes and transcription factors (TFs) in GBM and PD from an immune and inflammatory perspective, thus providing new insight into the comorbidity mechanism of these diseases. It is important to note that the conclusions require further clinical validation in the future. Additionally, the specific functions of the crosstalk genes need to be validated in vivo and in vitro. Subsequently, we plan to establish reliable animal models to further validate these findings, which will require significant time and funding to advance our theory. Nevertheless, this work remains essential and worth pursuing.

## Conclusion

In conclusion, we identified CXCR4, LY96, and C3 as key crosstalk genes in GBM and PD using multiple bioinformatics analysis methods. These genes may play a role in the crosstalk between these diseases through immune pathways. Furthermore, FLI1 was identified as a potential key transcription factor (TF) in IAs and PD. To our knowledge, this is the first study to report these findings. The current study provides new insights into the co-pathogenesis of GBM and PD, and further validation is required to confirm these results.

### Supplementary Information

Supplementary Figures.

Supplementary Table 1.

Supplementary Table 2.

Supplementary Table 3.

## Data Availability

The datasets analyzed in this study are available for download in the Gene Expression Omnibus (GEO) and TCGA-GBM dataset. GSE4290dataset: https://www.ncbi.nlm.nih.gov/geo/query/acc.cgi?acc=GSE4290. GSE10334 dataset: https://www.ncbi.nlm.nih.gov/geo/query/acc.cgi?acc=GSE10334. GSE12657 dataset: https://www.ncbi.nlm.nih.gov/geo/query/acc.cgi?acc=GSE12657. GSE16134 dataset: https://www.ncbi.nlm.nih.gov/geo/query/acc.cgi?acc=GSE16134. TCGA-GBM dataset: https://xenabrowser.net/datapages/?cohort=GDC%20TCGA%20Glioblastoma%20(GBM)&removeHub=https%3A%2F%2Fxena.treehouse.gi.ucsc.edu%3A443.
