# Supplementary material for: The potential crosstalk genes and molecular mechanisms between glioblastoma and periodontitis
Source: Sci Rep. 2024 Mar 12;14:5970. doi: 10.1038/s41598-024-56577-2 (PMC10933479; doi:10.1038/s41598-024-56577-2)
Supplement: Supplementary file 1 — Supplementary Figures. [file 41598_2024_56577_MOESM1_ESM.pdf]

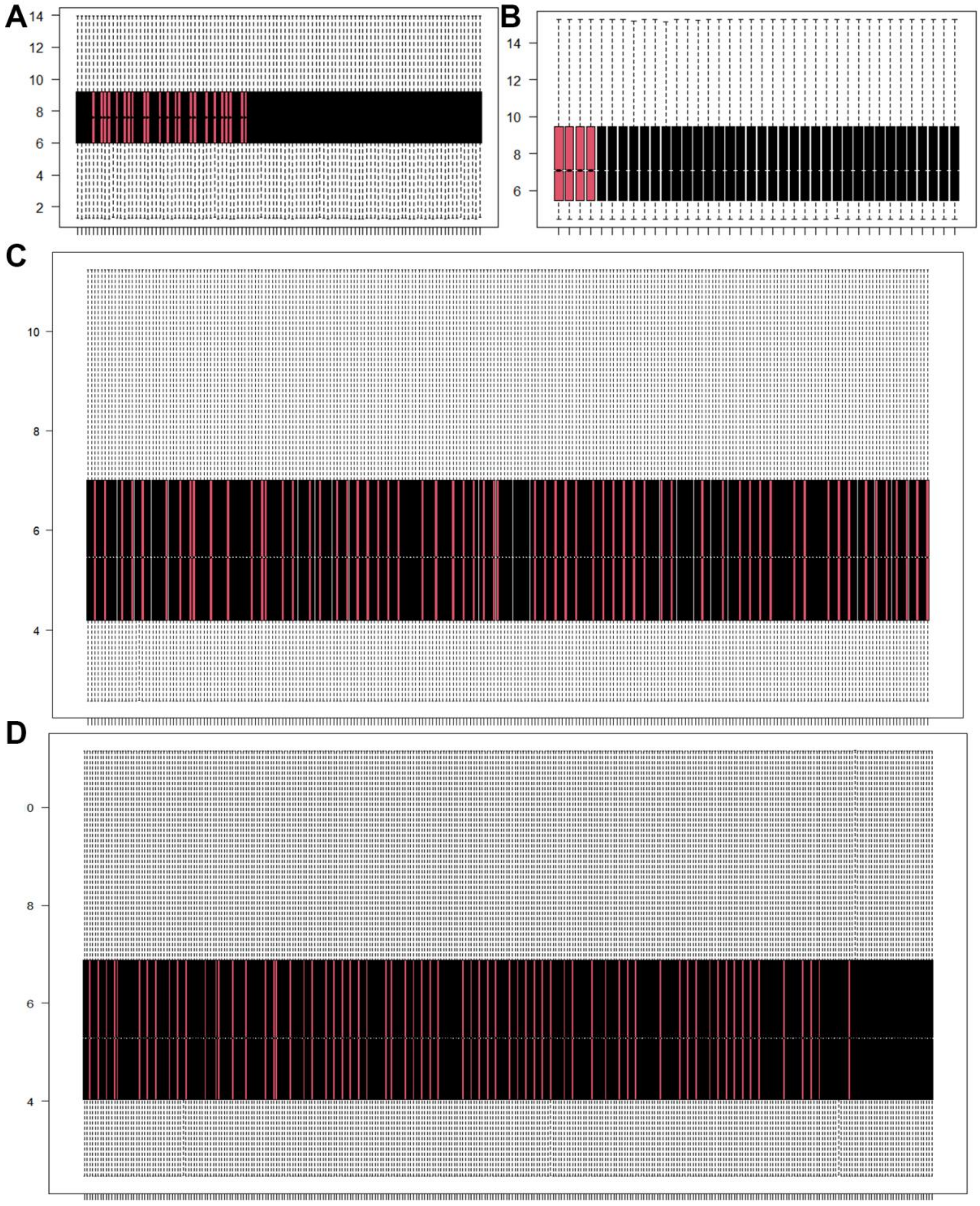

Figure 1. Normalization of the test set and external validation set used in this study. A-B. Normalization of the GBM datasets GSE4290 and GSE14805. C-D. Normalization of the periodontitis datasets GSE10334 and GSE16134.

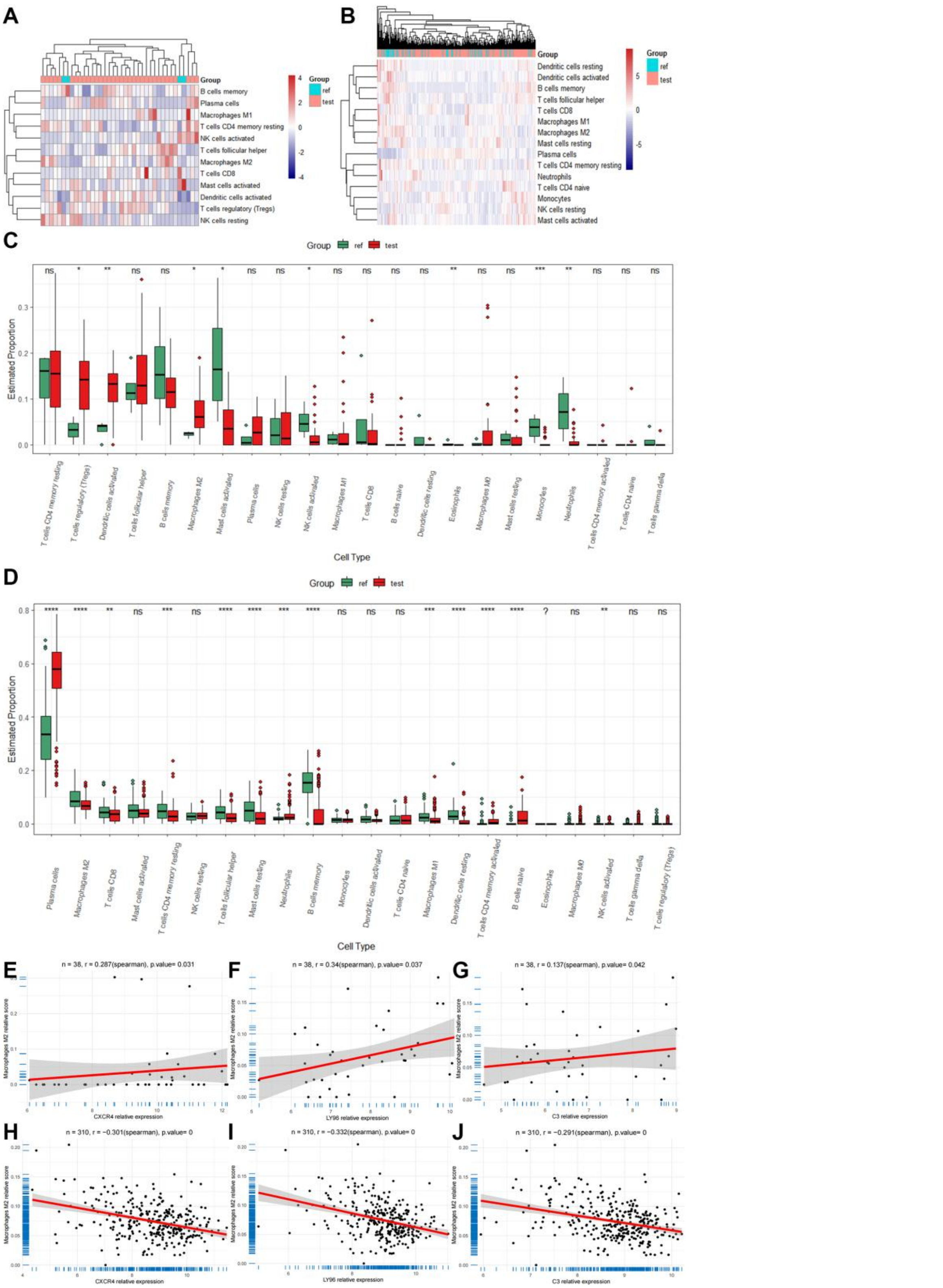

Figure 2. Immune infiltration analysis of the external validation dataset. A-B. Immune landscapes between datasets GSE14805 and GSE16134. C. Comparison of immune cell

composition between case and control groups in dataset GSE14805. D. Comparison of immune cell composition between case and control groups in dataset GSE16134. E. Significant positive correlation between the expression levels of CXCR4, LY96, and C3 and Macrophages M2 in dataset GSE14805. F. Significant negative correlation between the expression levels of CXCR4, LY96, and C3 and Macrophages M2 in dataset GSE16134. ****[1], $p < 0.00001$; ***, $p < 0.0001$; **, $p < 0.001$; *, $p < 0.01$.
